# Assessing the impact of novelty and conformity on hesitancy towards COVID-19 vaccines using mRNA technology

**DOI:** 10.1038/s43856-022-00123-6

**Published:** 2022-05-31

**Authors:** Ching Leong, Lawrence Jin, Dayoung Kim, Jeongbin Kim, Yik Ying Teo, Teck-Hua Ho

**Affiliations:** 1 Office of the Senior Deputy President and Provost, National University of Singapore, Singapore, Singapore; 2 Lee Kuan Yew School of Public Policy, National University of Singapore, Singapore, Singapore; 3 Global Asia Institute, National University of Singapore, Singapore, Singapore; 4 NUS Business School, National University of Singapore, Singapore, Singapore; 5 Saw Swee Hock School of Public Health, National University of Singapore, Singapore, Singapore

**Keywords:** Vaccines, Infectious diseases

## Abstract

**Background:**

Public hesitancy towards Covid-19 vaccines remains a major hurdle for mass vaccination programs today. While mRNA vaccines are more efficacious than conventional vaccines, it is unknown how much the novelty of this technology increases hesitancy.

**Methods:**

We quantify this “novelty penalty” in a large online experiment with 35,173 adults in nine countries. Subjects were randomly selected and assigned to one of two vaccine groups (conventional or mRNA), and one of five hypothetical inoculation rate groups (0%, 20%, 40%, 60%, or 80%). Subjects reported their willingness to accept the Covid-19 vaccine on a five-point Likert scale.

**Results:**

The novelty of the mRNA vaccine technology reduces the odds of a higher level of vaccine acceptance by 14.2% (odds ratio 0.858; *p* < 0.001). On the other hand, we find that social conformity reduces vaccine hesitancy. At a 0% inoculation rate, 31.7% report that they are “very likely” to get a mRNA vaccine while at a 20% inoculation rate, willingness jumps to 49.6%.

**Conclusions:**

The novelty of the mRNA vaccine increases hesitancy, but social conformity reduces it. A small group of early adopters can provide momentum for vaccination.

## Plain Language Summary

Vaccine hesitancy is a delay in acceptance or refusal of vaccines despite these being available. The hesitancy of the general public towards Covid-19 vaccines remains a challenge for mass vaccination programs. While Covid-19 vaccines using mRNA technology are more effective than vaccines produced using conventional technology, it is unknown how the novelty of the mRNA technology affects vaccine hesitancy among members of the public. Here, we surveyed over 35,000 adults from nine countries and found that the new mRNA vaccine technology leads to greater vaccine hesitancy. However, people are much more willing to accept the vaccine if at least 20% of the population has already received it. These findings show that while the novelty of the mRNA vaccine increases hesitancy, this hesitancy can be reduced by the presence of a small group of early adopters.

## Abstract (web summary)

Leong et al. surveyed more than 35,000 participants in nine countries to assess the impact of novelty and conformity on mRNA Covid-19 vaccine hesitancy. They show that the use of mRNA vaccine technology increases the odds of vaccine hesitancy significantly while social conformity reduces it.

## Introduction

Covid-19 has infected more than 235 million people and caused 4.8 million deaths worldwide^1^. The dominant public policy response has been to encourage mass vaccinations to allow healthcare systems and economies to return to normalcy. At efficacy rates of between 60% and 95%^2–4^, the vaccination rate required is estimated to be as high as 84% to 90% of the population^5^.

New vaccines have always encountered some degree of hesitancy among members of the public – past research has demonstrated this effect for vaccines for HPV and the influenza A (H1N1) virus, for example^6–10^. In the current pandemic, hesitancy towards vaccines for Covid-19 has been mapped across the world and remains a seemingly intractable obstacle in the fight against the pandemic^11–13^. In a survey of 13,426 people in nineteen countries, only 47% reported that they “completely agree” with getting a Covid-19 vaccine^14^. Indeed, in most countries, these levels of support were insufficient to meet the requirements for herd immunity^15^.

Recent studies have identified several determinants for Covid-19 vaccine acceptance, for example, acceptance was lower among females, people with lower levels of education, and in Black communities^16–18^. Vaccine acceptance is also influenced by the characteristics of the vaccine, such as efficacy, risk of serious side effects, the manufacturer, and the place of administration^16^.

Several recent studies investigated if people were more hesitant to get vaccines, which use novel, mRNA-based technology^19–21^. Results have been mixed, with a few studies finding some level of hesitancy towards new vaccine technologies among health care workers^19,20^, while one other study found no significant difference in preference between conventional (weakened viral) and mRNA-based vaccines for Covid-19^21^.

However, these studies were conducted prior to the approval and roll-out of Covid-19 vaccines to the public (which began with the approval of Pfizer-BioNTech’s mRNA-based Covid-19 vaccine for emergency use in the United States of America on December 11, 2020). Public interest in mRNA vaccine technology jumped around the second week of December when the web search volume for “mRNA vaccine” increased more than 30-fold^22^. The rise in interest was accompanied by substantial misinformation regarding the new vaccine technology^23^, which in turn significantly increased vaccine hesitancy^24^.

It is both urgent and important to add empirical heft to our understanding of public hesitancy towards conventional and mRNA vaccines for Covid-19 at a crucial moment when information about vaccines is spreading rapidly and many people have experienced receiving the vaccines.

This study has two main goals. The first is to determine the increased hesitancy towards novel mRNA vaccines after the vaccines were approved for public use. We do this by conducting a large-scale, global survey to investigate if there are differences in willingness to accept conventional and mRNA-based vaccines. We anticipate an increase in vaccine hesitancy due to the novelty of the mRNA vaccine technology, which we call a “novelty penalty.” This term first coined by psychologists to refer to people’s preference for familiar experiences over novel ones^25^.

Our second goal is to investigate forces that may reduce vaccine hesitancy. Previous research has shown that social conformity is useful in overcoming hesitancy towards novel science^26^. Hence, we examine how an individual’s decision to accept the vaccine may be influenced by the decisions of others in the community. Specifically, would a person’s willingness to get vaccinated increase dramatically if the person found out that many members of the community had already been vaccinated? What level of vaccination would be required to trigger this dramatic increase? Such bandwagon effects, if proven, will provide governments with a powerful policy instrument against the pandemic.

We find that the novelty of the mRNA vaccine technology reduces the odds of a higher level of vaccine acceptance by 14.2% (odds ratio 0.858; *p* < 0.001). The magnitude of this “novelty penalty” varies across countries. We also find that social conformity reduces vaccine hesitancy. When no one in the country is vaccinated, only 31.7% of people responded that they are very likely to get an mRNA vaccine for Covid-19 and 35.1% a conventional vaccine. Upon learning that 20% of their peers have been vaccinated, the proportion jumps to 49.6% for an mRNA vaccine and 52.4% for a conventional vaccine. Above 20%, the proportion of people responding that they are very likely to receive the vaccine continues to increase, although more slowly. Our findings highlight the importance of early adopters to create momentum for vaccinations.

## Methods

### Experimental Design

Between February 3 and March 5, 2021, we surveyed 35,180 adults in nine of the most populated countries in the Americas (Brazil, Mexico, and the United States of America), Asia (China, India, and Indonesia) and Europe (Germany, Russia, and the United Kingdom)^27^. Around 3900 subjects were recruited in each country. The survey implementation was administered by SurveyMonkey, a third-party survey company, and the recruitment of online subjects was conducted by one of SurveyMonkey’s global panel partners. SurveyMonkey implemented its proprietary method of quota sampling to meet the age and gender distributions of each country’s census. The only inclusion criterion was that the subjects had to be 18 years of age or older. The National University of Singapore’s Institutional Review Board (NUS-IRB) granted the study (NUS-IRB-2020-733) an exemption from IRB review and from the need for informed consent, as it was deemed to be of minimal risk and did not involve the collection or use of any potentially sensitive data. Following best practice in the behavioral sciences, the study was also pre-registered at ClinicalTrials.gov (NCT04693689).

The survey asked about people’s confidence in vaccines in general (based on four questions used in the Wellcome Global Monitor study^28^), and their willingness to receive a Covid-19 vaccine if it were provided for free. In addition, we examined the extent to which their willingness to receive a Covid-19 vaccine was influenced by two factors: the novelty of mRNA vaccine technology, and the hypothetical vaccine adoption rate in their country. All subjects were adults (aged 18 and above) and received a small amount of financial compensation for participating in the survey.

To study if Covid-19 vaccine acceptance is influenced first, by the type of technology, and second, by the vaccine adoption rate in the country, we implemented a 2 × 5, between-subjects randomized control trial (RCT) design. We provided a brief description of how conventional and mRNA-based vaccines work and then elicited the subjects’ willingness to get vaccinated using two technologies (conventional or mRNA) under five hypothetical vaccine adoption rates (0%, 20%, 40%, 60%, or 80%). The full survey instrument is available in Supplementary Methods.

The key outcome of interest was the response to the question, “Suppose the [conventional/RNA] COVID-19 vaccine is endorsed by your Government, free, [but no one/and 20%/and 40%/and 60%/and 80% of people] in your country [has/have] received the vaccine. How likely are you to accept the vaccination?” Five answer choices were given (“Very likely,” “Somewhat likely,” “Neither likely nor unlikely,” “Somewhat unlikely,” “Very unlikely”). Use of the 5-point Likert scale was consistent with previous large-scale research on vaccine acceptance^9,10,28^. Prior research suggests that people usually only carry out the specific behavior in question if their intentions are at least mildly positive^29^. We therefore focused on those “very likely” to accept the vaccine as a reliable predictor of actual vaccination rates. (In Supplementary Discussion, we report results using the proportion of people who reported that they were either “very likely” or “somewhat likely” to accept the vaccine. These results likely overstate the actual vaccination rate).

### Statistics and Reproducibility

To test the “novelty penalty” hypothesis, we conducted a Kolmogorov-Smirnov test. This was done to detect whether there was a significant difference in the distribution of responses to the vaccine acceptance question regarding conventional and mRNA vaccines. In addition, we conducted a two-sample proportion test to check whether there is a difference in the proportion of respondents very likely to accept a conventional vaccine and those very likely to accept an mRNA vaccine.

To check whether there was an interaction between vaccine novelty and the adoption rate, we conducted a two-way analysis of variance (ANOVA). We also conducted a multivariate ordered logistic regression analysis to estimate the effects of vaccine novelty and the adoption rate on the odds of subjects having a higher level of vaccine hesitancy. All analyses were performed using Stata software version 13.0. The raw data is included as Supplementary Data 1^30^. The analytic code is included as Supplementary Data 2^30^.

### Reporting summary

Further information on research design is available in the Nature Research Reporting Summary linked to this article.

## Results

In total, 35,180 subjects responded to the survey. Seven responses were dropped due to invalid entries for age, leaving a final sample size of 35,173. Summary statistics of responses are shown in Supplementary Data 4. The average age of respondents was 39.2, and 50.0% of respondents were female. About 61.6% of respondents received more than 12 years of formal schooling.

First, we asked questions relating to general attitudes towards vaccines: 44.2% strongly agreed that vaccines are safe; 45.1% strongly agreed that vaccines are effective, and 59.6% strongly agreed that vaccines are important for children to take. 76.3% reported that it is very necessary for a friend’s child to be vaccinated. There were no statistically significant differences in general attitudes towards vaccines among the 10 treatment groups.

However, there were significant differences in attitudes towards Covid-19 vaccines that use conventional vaccine technology versus those that use mRNA: 75.1% believed that a conventional vaccine should be entirely subsidized by the government compared to only 71.2% for an mRNA vaccine (two-sample proportions test; *z* = 8.375, *p* < 0.001, Cohen’s *h* = 0.089).

Next, we asked three questions relating to subjects’ willingness to accept Covid-19 vaccines. First, we asked for the likelihood of Covid-19 vaccine acceptance before showing a hypothetical adoption rate in the country; second, we asked the same question after randomly showing one of five hypothetical adoption rates in the country (0%, 20%, 40%, 60%, 80%); and finally, we asked the likelihood of subjects recommending getting a Covid-19 vaccine to their loved ones.

To the first question (asked prior to being shown an adoption rate), 58.7% of respondents said they were very likely to accept a conventional vaccine for Covid-19, compared to 52.7% for an mRNA vaccine (two-sample proportions test; *z* = 11.274, *p* < 0.001, Cohen’s *h* = 0.120). The difference of six percentage points is statistically significant.

To the second question (asked after being shown a hypothetical adoption rate; this question is the main dependent variable of interest), on average, 52.1% reported that they were very likely to accept a conventional vaccine for Covid-19, compared to 48.7% for an mRNA vaccine (two-sample proportions test; *z* = 6.430, *p* < 0.001, Cohen’s *h* = 0.069) The 3.4 percentage-point difference, while statistically significant, is smaller in magnitude compared to the first question. (It is likely that this smaller magnitude is due to the implicit assumption in the first question that there is already some level of adoption within the country).

A two-way analysis of variance (ANOVA) was conducted to test a potential interaction effect between the vaccine technology and the vaccine adoption rate in each country. There was a significant difference in the proportion of people very likely to get vaccinated, between mRNA vaccines and conventional vaccines (*F*(1,35163) = 41.64, *p* < 0.001), as well as among adoption rates (*F*(4,35163) = 288.42, *p* < 0.001). There was no interaction effect between the two treatment variables (*F*(4,35163) = 0.07, *p* = 0.990).

To the third question, 50.4% said they were very likely to recommend getting a conventional Covid-19 vaccine to their loved ones, compared to 46.6% for an mRNA vaccine (two-sample proportions test; *z* = 6.985, *p* < 0.001, Cohen’s *h* = 0.075).

**Fig. 1:**
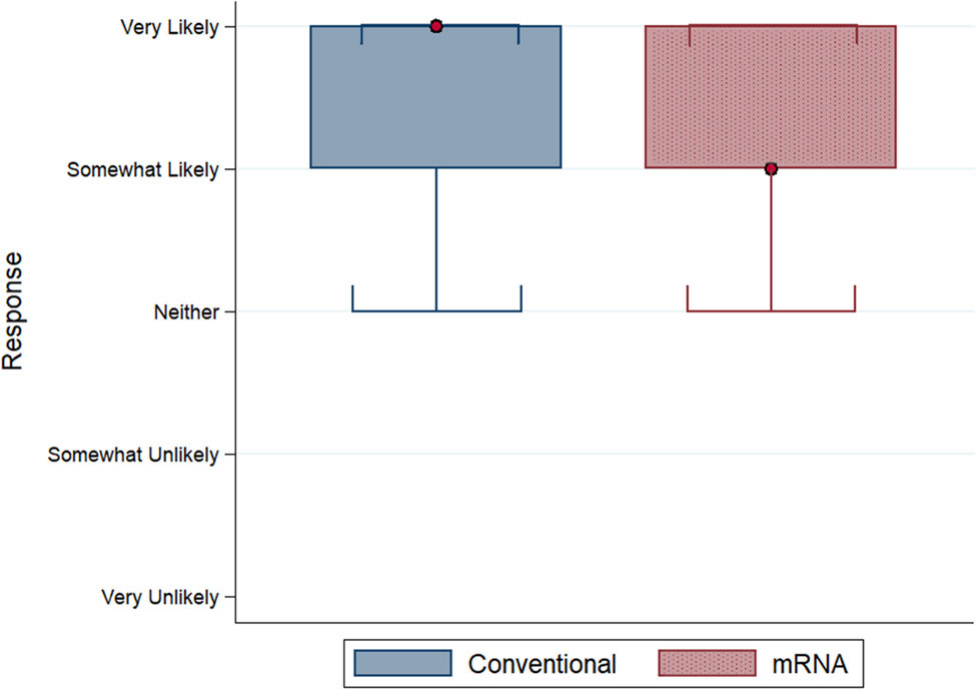
The distribution (box and whisker plots) of responses, by vaccine type, to the question “Suppose the [conventional/RNA] Covid-19 vaccine is endorsed by your Government, free, [but no one/and 20%/and 40%/and 60%/and 80% of people] in your country [has/have] received the vaccine. How likely are you to accept the vaccination?” Boxes indicate median and interquartile range (IQR) with the red dot specifying median value, and whiskers indicate range (data within 1.5*IQR). Conventional vaccine *n* = 17,527 responses; mRNA vaccine *n* = 17,646 responses. The source dataset for the figure can be found in Supplementary Data 1.

Figure 1 shows the distribution of responses to the Covid-19 vaccine acceptance question (the second of the three questions above). The two distributions are significantly different from each other (Kolmogorov-Smirnov test; *D* = 0.034, *p* < 0.001). A total of 79.5% indicated that they were “very likely” and “somewhat likely” to accept a conventional Covid-19 vaccine compared to 77.0% for an mRNA vaccine (two-sample proportions test; *z* = 5.584, *p* < 0.001, Cohen’s *h* = 0.060). We find smaller differences between adopting mRNA and conventional vaccines when we total “very likely” and “somewhat likely” and use the sum as a predictor of actual vaccination rates.

The respondents’ acceptance of a Covid-19 vaccine was also positively correlated with their attitudes towards vaccines in general. The correlation between Covid-19 vaccine acceptance and strong agreement that (1) vaccines are “safe” was 0.422 (*p* < 0.001); (2) vaccines are “effective” was 0.393 (*p* < 0.001); (3) vaccines are “important for children to have” was 0.372 (*p* < 0.001); and (4) it is “necessary for your friend to vaccinate their child” was 0.368 (*p* < 0.001). The positive relationship between Covid-19 vaccine acceptance and general attitudes towards vaccines was also observed in each country (Supplementary Fig. 1).

**Fig. 2:**
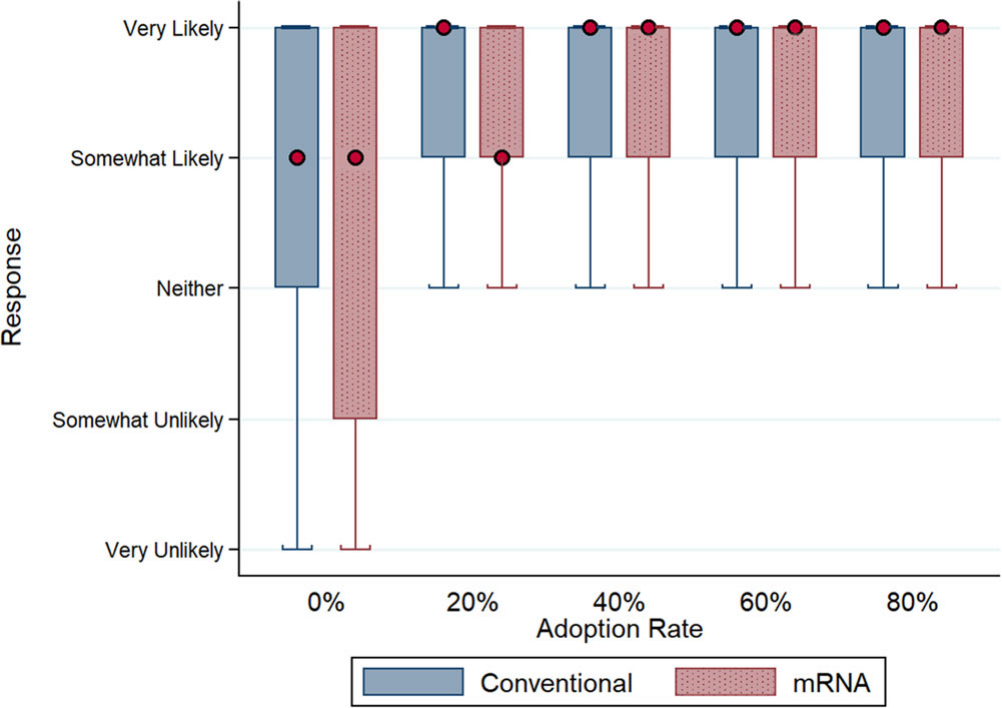
The distribution (box and whisker plots) of responses, by vaccine type and adoption rate, to the question “Suppose the [conventional/RNA] Covid-19 vaccine is endorsed by your Government, free, [but no one/and 20%/and 40%/and 60%/and 80% of people] in your country [has/have] received the vaccine. How likely are you to accept the vaccination?” Boxes indicate median and interquartile range (IQR) with the red dot specifying median value, and whiskers indicate range (data within 1.5*IQR). Conventional vaccine *n* = 17,527 responses (0% adoption rate *n* = 3,488 responses, 20% *n* = 3,551 responses, 40% *n* = 3,620 responses, 60% *n* = 3,488 responses, 80% *n* = 3,380 responses); mRNA vaccine *n* = 17,646 responses (0% adoption rate *n* = 3,548 responses, 20% *n* = 3,648 responses, 40% *n* = 3,520 responses, 60% *n* = 3,514 responses, 80% *n* = 3,416 responses). The source dataset for the figure can be found in Supplementary Data 1.

We find that a small group of early adopters can significantly boost people’s willingness to get vaccinated (Fig. 2). When the vaccine adoption rate is zero, 35.1% of people responded that they are very likely to get a conventional vaccine and 31.7% an mRNA vaccine. At a 20% adoption rate, the proportion jumps to 52.4% for a conventional vaccine, and 49.6% for an mRNA vaccine. The relationship between the adoption rate and vaccine acceptance remains positive beyond a 20% adoption rate, but the increase is more gradual. At an 80% adoption rate, 60.8% are very likely to get a conventional vaccine and 57.3% an mRNA vaccine. This pattern was similar for all the countries surveyed.

We estimate a multivariate ordered logistic regression model to test for the effects of the two key independent variables of interest – novelty of the mRNA vaccine technology and the vaccine adoption rate – on the respondents’ odds of having a higher level of vaccine acceptance. The dependent variable is vaccine acceptance, which takes on five values from 0 (“Very unlikely”) to 4 (“Very likely”). The independent variables include an indicator for mRNA vaccine technology, indicators for vaccine adoption rates (20%, 40%, 60%, 80%; baseline is 0%), as well as the respondent’s gender (male, female, other), age (in years), years of schooling (7–9, 10–12, >12; baseline is ≤ 6), and country of residence (the baseline is the USA).

The results are shown in Table 1. The estimated coefficient for mRNA indicator is −0.153 (odds ratio 0.858, *p* < 0.001), implying that the novel vaccine technology reduces the odds by 14.2%. The estimate is highly statistically significant. Consistent with our earlier findings (Fig. 2), we observe a large and significant increase in vaccine acceptance when the adoption rate moves from 0% to 20% (odds ratio 2.321, *p* < 0.001), but beyond 20%, the increases are more gradual. The results suggest the importance of early adopters in creating momentum for vaccination rates.

**Table 1:** Ordered Logistic Regression Results.

	(1) Dependent variable: Vaccine acceptance
mRNA	−0.153*** (0.021) [OR=0.858]
Adoption rate (Baseline: 0%)	
20%	0.842*** (0.032) [OR=2.321]
40%	0.948*** (0.032) [OR=2.581]
60%	1.024*** (0.032) [OR=2.784]
80%	1.186*** (0.033) [OR=3.276]
Female	−0.278*** (0.021) [OR=0.758]
Age	0.015*** (0.001) [OR=1.016]
Years of schooling (Baseline: ≤6)	
7–9	−0.056 (0.053) [OR=0.945]
10–12	−0.067 (0.044) [OR=0.935]
>12	0.177*** (0.041) [OR=1.193]
Country (Baseline: USA)	
Brazil	1.024*** (0.047) [OR=2.784]
China	0.818*** (0.045) [OR=2.266]
Germany	0.038 (0.044) [OR=1.039]
India	0.810*** (0.044) [OR=2.247]
Indonesia	0.718*** (0.046) [OR=2.049]
Mexico	1.099*** (0.047) [OR=3.002]
Russia	−0.617*** (0.044) [OR=0.540]
UK	0.619*** (0.045) [OR=1.856]
Pseudo *R*^2^	0.051
Observations	35,173

Notes: Table reports estimates from multivariate ordered logistic regression model. The dependent variable is vaccine acceptance, which takes on five values (0 = “Very unlikely”, …, 4 = “Very likely”). Estimated coefficients are shown, with standard errors in parentheses and odds ratios in square brackets. * *p* < 0.005 ** *p* < 0.001 *** *p* < 0.0001.

We also observe significant differences in vaccine acceptance levels across the respondents’ demographic variables. On average, females have lower vaccine acceptance levels (odds ratio 0.758, *p* < 0.001). There is also a positive relationship between age and the willingness to accept the Covid-19 vaccine (odds ratio 1.016, *p* < 0.001). On average, each additional year of age was associated with 1.6% increase in the odds of higher vaccine acceptance, holding other covariates constant in the model. This is consistent with previous findings^16^, and may be due to the vulnerability of older subjects to the disease (see Supplementary Fig. 2 for the distribution of responses by age group). Education also mattered; subjects with more than 12 years of education had significantly higher odds compared to others (vs. baseline group of under 6 years of education: the odds ratio is 1.193, *p* < 0.001; vs. 7–9 years of education: *p* < 0.001; vs. 10–12 years of education: *p* < 0.001) (see Supplementary Fig. 3 for the distribution of responses by years of schooling).

There were significant differences in vaccine acceptance across the nine countries. Subjects in Mexico exhibited the highest level of vaccine acceptance, with three times the odds compared to the United States (odds ratio 3.002, *p* < 0.001), followed by Brazil (odds ratio 2.784, *p* < 0.001), China (odds ratio 2.266, *p* < 0.001), India (odds ratio 2.247, *p* < 0.001), Indonesia (odds ratio 2.049, *p* < 0.001), UK (odds ratio 1.856, *p* < 0.001), and Germany (odds ratio 1.039, *p* = 0.384). Russia had the lowest odds among the nine countries, 46.0% less than the United States (odds ratio 0.540, *p* < 0.001).

When we focus on the subjects who are very likely to accept the vaccine, the highest proportions were observed in Brazil, Mexico, the UK, India, and Indonesia. In Brazil, 64.2% were very likely to accept a conventional vaccine and 58.8% an mRNA vaccine. In Mexico, the results were 61.9% for a conventional vaccine and 59.7% for an mRNA vaccine. In the UK, the results were 58.0% for a conventional vaccine and 54.0% for an mRNA vaccine. In India, the results were 54.9% for a conventional vaccine and 52.0% for an mRNA vaccine. And in Indonesia the results were 55.1% for a conventional vaccine and 51.0% for an mRNA vaccine.

The lowest vaccine acceptance was observed in Russia, Germany, the USA, and China. In Russia, only 31.1% were very likely to accept a conventional vaccine for Covid-19, and 26.3% an mRNA vaccine. In Germany, the results were 43.1% for a conventional vaccine and 40.5% for an mRNA vaccine. In the United States, the results were 46.6% for a conventional vaccine and 43.9% for an mRNA vaccine. And in China, the results were 53.2% for a conventional vaccine and 50.9% for an mRNA vaccine. Supplementary Data 5 provides a full breakdown of the attitudes towards conventional and mRNA vaccines for each country.

We found no significant difference in the magnitude of the novelty penalty across gender, age, and the adoption rate in the country (Supplementary Data 6). We observe heterogeneity in the magnitude of the novelty penalty across the nine countries. The novelty penalty was largest in Russia, where it was found that the novelty of the technology reduces the odds of higher vaccine acceptance by 24.2% (with respect to the acceptance rate for conventional vaccines in that country) (odds ratio 0.758, *p* < 0.001), followed by Brazil (odds ratio 0.776, *p* < 0.001), the UK (odds ratio 0.843, *p* = 0.008), the USA (odds ratio 0.852, *p* = 0.011), Indonesia (odds ratio 0.864, *p* = 0.020) and China (odds ratio 0.880, *p* = 0.033). The smallest novelty penalty was observed in Mexico (odds ratio 0.962, *p* = 0.557), India (odds ratio 0.903, *p* = 0.066) and Germany (odds ratio 0.891, *p* = 0.054). Supplementary Table 1 provides *p*-values for the difference in novelty penalty between countries.

We also investigated whether the novelty penalty was associated with how severe the outbreak is locally. We used three proxies for the outbreak severity: the cumulative number of Covid-19 cases per population (Supplementary Fig. 4A), the case-fatality ratio (Supplementary Fig. 4B), and the cumulative number of Covid-19 deaths per 10,000 population (Supplementary Fig. 4C). We found no association between the novelty penalty and outbreak severity, as measured by the three proxies (Supplementary Data 3).

## Discussion

Our study is among the very few large global surveys conducted immediately after vaccination drives began in the current pandemic. We empirically estimate increased public hesitancy towards novel mRNA vaccines compared to conventional vaccines. Of the 35,173 respondents, 52.1% said they were “very likely” to get a conventional vaccine, compared to 48.7% for an mRNA vaccine. The novelty penalty of three percentage points is highly statistically significant. In a multivariate analysis, we find that the novelty of the mRNA vaccine technology reduces the odds of having a higher level of vaccine acceptance by 14.2%.

We find evidence of the impact of conformity in people’s willingness to get vaccinated. Upon learning that 20% of their peers (instead of 0%) have been vaccinated, a significantly larger proportion of people indicated that they were “very likely” to get vaccinated. Above 20%, the proportion of people continues to increase, albeit more slowly. At an 80% adoption rate, 60.8% of people indicated they were “very likely” to get for a conventional vaccine and 57.3% indicated the same for an mRNA vaccine. Hence, social conformity is one way to reduce vaccine hesitancy. There is, however, no interaction effect between conformity and the novelty penalty – that is, conformity does not reduce the novelty penalty, and even at an 80% uptake level, there remains a significant penalty.

Our findings suggest that herd immunity achieved through vaccinations is likely to be regional for two reasons. First, our study shows that the overall proportion of people indicating they are “very likely” to get vaccinated is around 50%, well below the 84% to 90% level needed for herd immunity. However, our study was conducted at the start of vaccination programs when people were still cautious about vaccines. Now that the programs have proven highly successful, especially in developed countries, we expect a significant increase in the proportion of people “very likely” to get vaccinated. Second, our study shows that the difference between people’s willingness to get the mRNA vaccine is only 3.4% lower than their willingness to get the conventional vaccine. Given the much higher level of efficacy of an mRNA vaccine (94–95%) when compared to a conventional vaccine (below 70%), herd immunity achieved through vaccinations may be more likely in countries offering an mRNA vaccine ^2–4^.

Our study bears three limitations. First, the degree of the novelty penalty observed in our survey was likely influenced by the way we described vaccine technologies to the respondents. In designing the survey, we reviewed scientific publications on Covid-19 to ensure that our written communications to the subjects were concise, accurate and objective. However, we recognize that different descriptions of the vaccines can elicit different responses. One area for future research may be to investigate how different descriptions and framing (e.g., written with more or less emotional language) may influence the level of the novelty penalty. Second, we have little empirical evidence on how survey responses translate into actual vaccine take-up behavior. In our study, we focused on the “very likely” responses as a conservative predictor of actual vaccine uptake rates. Future research can examine how different response categories map to actual vaccination behavior. Third, we measure vaccine hesitancy when vaccination programs had just started. Ideally, one would want to measure vaccine hesitancy across time as more information on the vaccine becomes available. Such a panel study would be costly but a worthy endeavor as governments prepare for the next pandemic.

## Supplementary information

Description of Additional Supplementary Files

Supplementary Data 1

Supplementary Data 2

Supplementary Data 3

Supplementary Data 4

Supplementary Data 5

Supplementary Data 6

Reporting Summary

Supplementary Information

## Data Availability

All the datasets, including the source data, have been deposited in OSFHome and can be accessed without restriction at https://osf.io/6fuvp^30^. The raw data are available as Supplementary Data 1. The data for supplementary figure 4 are available as Supplementary Data 3. The summary statistics are available as Supplementary Data 4. The data on attitudes towards conventional and mRNA vaccines are available as Supplementary Data 5. And the data on the heterogeneity of the novelty penalty across demographics are available as Supplementary Data 6.

## Code availability

The code to analyse the data has been deposited in OSFHome and can be accessed without restriction at https://osf.io/6fuvp^30^.
