# Supplementary material for: Assessing the impact of novelty and conformity on hesitancy towards COVID-19 vaccines using mRNA technology
Source: Commun Med (Lond). 2022 May 31;2:61. doi: 10.1038/s43856-022-00123-6 (PMC9156695; doi:10.1038/s43856-022-00123-6)
Supplement: Supplementary file 1 — Description of Additional Supplementary Files [file 43856_2022_123_MOESM1_ESM.pdf]

Description of Additional Supplementary Files

File Name: Supplementary Data 1

Description: Raw Data

File Name: Supplementary Data 2

Description: Code

File Name: Supplementary Data 3

Description: Data for Supplementary Figure 4

File Name: Supplementary Data 4

Description: Summary Statistics

File Name: Supplementary Data 5

Description: Attitudes Towards Conventional and mRNA Vaccines, by Country

File Name: Supplementary Data 6

Description: Heterogeneity of Novelty Penalty across Demographics
