# Supplementary material for: Assessing the impact of novelty and conformity on hesitancy towards COVID-19 vaccines using mRNA technology
Source: Commun Med (Lond). 2022 May 31;2:61. doi: 10.1038/s43856-022-00123-6 (PMC9156695; doi:10.1038/s43856-022-00123-6)
Supplement: Supplementary file 8 — Reporting Summary [file 43856_2022_123_MOESM8_ESM.pdf]

nature research

Corresponding author(s): Teck-Hua Ho

Last updated by author(s): May 4, 2022

Reporting Summary

Nature Research wishes to improve the reproducibility of the work that we publish. This form provides structure for consistency and transparency in reporting. For further information on Nature Research policies, see our Editorial Policies and the Editorial Policy Checklist.

Statistics

For all statistical analyses, confirm that the following items are present in the figure legend, table legend, main text, or Methods section.

n/a	Confirmed	
☐	☒	The exact sample size (n) for each experimental group/condition, given as a discrete number and unit of measurement
☐	☒	A statement on whether measurements were taken from distinct samples or whether the same sample was measured repeatedly
☐	☒	The statistical test(s) used AND whether they are one- or two-sided<br>*Only common tests should be described solely by name; describe more complex techniques in the Methods section.*
☐	☒	A description of all covariates tested
☒	☐	A description of any assumptions or corrections, such as tests of normality and adjustment for multiple comparisons
☐	☒	A full description of the statistical parameters including central tendency (e.g. means) or other basic estimates (e.g. regression coefficient) AND variation (e.g. standard deviation) or associated estimates of uncertainty (e.g. confidence intervals)
☐	☒	For null hypothesis testing, the test statistic (e.g. F, t, r) with confidence intervals, effect sizes, degrees of freedom and P value noted<br>*Give P values as exact values whenever suitable.*
☒	☐	For Bayesian analysis, information on the choice of priors and Markov chain Monte Carlo settings
☒	☐	For hierarchical and complex designs, identification of the appropriate level for tests and full reporting of outcomes
☐	☒	Estimates of effect sizes (e.g. Cohen's d, Pearson's r), indicating how they were calculated

Our web collection on statistics for biologists contains articles on many of the points above.

Software and code

Policy information about availability of computer code

Data collection	Data was collected by a major online survey company (SurveyMonkey).
Data analysis	Data analysis was conducted using Stata software version 13.

For manuscripts utilizing custom algorithms or software that are central to the research but not yet described in published literature, software must be made available to editors and reviewers. We strongly encourage code deposition in a community repository (e.g. GitHub). See the Nature Research guidelines for submitting code & software for further information.

Data

Policy information about availability of data

All manuscripts must include a data availability statement. This statement should provide the following information, where applicable:

- Accession codes, unique identifiers, or web links for publicly available datasets
- A list of figures that have associated raw data
- A description of any restrictions on data availability

All the datasets, including the source data, have been deposited in OSFHome and can be accessed without restriction at https://osf.io/6fuvp.30 The raw data are available as Supplementary Data 1. The data for supplementary figure 4 are available as Supplementary Data 3. The summary statistics are available as Supplementary Data 4. The data on attitudes towards conventional and mRNA vaccines are available as Supplementary Data 5. And the data on the heterogeneity of the novelty penalty across demographics are available as Supplementary Data 6.

Field-specific reporting

Please select the one below that is the best fit for your research. If you are not sure, read the appropriate sections before making your selection.

☐ Life sciences ☒ Behavioural & social sciences ☐ Ecological, evolutionary & environmental sciences

For a reference copy of the document with all sections, see nature.com/documents/nr-reporting-summary-flat.pdf

Behavioural & social sciences study design

All studies must disclose on these points even when the disclosure is negative.

Study description	This study uses quantitative experimental dataset obtained from an online survey experiment. The study investigates factors that influence Covid-19 vaccine hesitancy, including the novelty of the mRNA vaccine technology and the vaccination decisions of others in the community.
Research sample	We conduct an online experiment involving 35,180 adults in nine countries. The subjects were randomly sampled by the online survey platform (SurveyMonkey) to be representative of the population in each of the nine countries.
Sampling strategy	Stratified random sampling was conducted by SurveyMonkey to ensure that the participating subjects' age and gender were representative of the populations of nine countries. A larger sample size target of 3,600 subjects per country were determined based on prior online surveys that were conducted on attitudes towards Covid-19 vaccines.
Data collection	Data collection was completed by SurveyMonkey, a major online survey platform.
Timing	Data was collected between February 3 to March 5, 2021.
Data exclusions	The only inclusion criterion was having an age of 18 or above. No other exclusion criteria were used. Once the data was collected, 7 observations were excluded from the analysis sample because the data entered for age was invalid.
Non-participation	A single online survey was administered through an online survey platform (SurveyMonkey). The survey completion rate was 85%. The researchers do not have access to the survey response rate (i.e., the number of people who chose to participate divided by the number of people who were contacted to participate in the survey.)
Randomization	Subjects were randomly assigned to experimental groups within the SurveyMonkey online survey platform.

Reporting for specific materials, systems and methods

We require information from authors about some types of materials, experimental systems and methods used in many studies. Here, indicate whether each material, system or method listed is relevant to your study. If you are not sure if a list item applies to your research, read the appropriate section before selecting a response.

Materials & experimental systems

n/a	Involved in the study	
☒	☐	Antibodies
☒	☐	Eukaryotic cell lines
☒	☐	Palaeontology and archaeology
☒	☐	Animals and other organisms
☐	☒	Human research participants
☒	☐	Clinical data
☒	☐	Dual use research of concern

Methods

n/a	Involved in the study	
☒	☐	ChIP-seq
☒	☐	Flow cytometry
☒	☐	MRI-based neuroimaging

Human research participants

Policy information about studies involving human research participants

Population characteristics	Data from 35,173 adults was analyzed for the study. The average age of subjects was 39 years old, and 50% were female.
Recruitment	Subjects were recruited by a major online survey company (SurveyMonkey) using the quota sampling method to ensure that the participating subjects' ages and genders were representative of the populations of the nine countries.
Ethics oversight	The National University of Singapore’s Institutional Review Board (NUS-IRB) granted the study (NUS-IRB-2020-733) an exemption from IRB review and from the need for informed consent, as it was deemed to be of minimal risk and did not involve the collection or use of any potentially sensitive data.

Note that full information on the approval of the study protocol must also be provided in the manuscript.
