# Supplementary material for: Assessing the impact of novelty and conformity on hesitancy towards COVID-19 vaccines using mRNA technology
Source: Commun Med (Lond). 2022 May 31;2:61. doi: 10.1038/s43856-022-00123-6 (PMC9156695; doi:10.1038/s43856-022-00123-6)
Supplement: Supplementary file 9 — Supplementary Information [file 43856_2022_123_MOESM9_ESM.docx]

**Assessing the impact of novelty and conformity on hesitancy towards**

**COVID-19 vaccines using mRNA technology**

Supplementary Materials

Ching Leong^1^, Lawrence Jin^2^, Dayoung Kim^3^, Jeongbin Kim^4^, Yik Ying Teo^5^, Teck-Hua Ho^1^*

^1^ Office of the Senior Deputy President and Provost, National University of Singapore, Singapore. ^2^ Lee Kuan Yew School of Public Policy, National University of Singapore, Singapore. ^3^ Global Asia Institute, National University of Singapore, Singapore. ^4^ NUS Business School, National University of Singapore, Singapore. ^5^ Saw Swee Hock School of Public Health, National University of Singapore, Singapore. *e-mail: [teck@nus.edu.sg](mailto:teck@nus.edu.sg)

**Contents**

1. Supplementary Methods
2. Supplementary Discussion
3. Supplementary References

**Supplementary Methods**

Survey instrument: Questionnaire on attitudes towards COVID-19 vaccines using mRNA technology

Welcome!

This survey is part of a research study. The purpose of this research is to better understand the perception of vaccines (substances that prevent the spread of disease) and the human decision-making process. Any personal data will remain strictly confidential and be used for this research purpose only.

1. On the following three statements, please rate to what extent you agree or disagree on a five-point scale.

1.1. Vaccines are safe.

- - - Strongly agree
    - Somewhat agree
    - Neither agree nor disagree
    - Somewhat disagree
    - Strongly disagree

1.2. Vaccines are effective.

- Strongly agree
- Somewhat agree
- Neither agree nor disagree
- Somewhat disagree
- Strongly disagree

1.3. Vaccines are important for children to have.

- Strongly agree
- Somewhat agree
- Neither agree nor disagree
- Somewhat disagree
- Strongly disagree

1. Suppose you have a friend who has a child. To what extent do you think it is necessary for your friend to vaccinate the child to prevent against childhood diseases such as polio, measles, or tuberculosis?

- Very necessary
- Somewhat necessary
- Neither necessary nor unnecessary
- Somewhat unnecessary
- Very unnecessary

We would now like to ask you about your thoughts regarding vaccines for COVID-19.

There are a number of ways in which vaccines help you gain immunity to a virus. In essence, the body’s immune system needs to be exposed to a component of the virus (the antigen) so that immunity develops.

In conventional vaccines, weakened or inactive virus is injected into your body to invoke an immune response.

A new type of vaccine that is being developed is called an RNA vaccine. RNA vaccines inject RNA molecules into your body, and these molecules provide instructions for your cells to produce a component of the virus, which in turn invokes an immune response. So, no actual virus is injected into the body. However, prior to COVID-19, no RNA vaccine had been approved for human use on a large scale.

Both conventional vaccines and RNA vaccines are currently being developed for the COVID-19 virus. The conventional vaccine has been widely used to eradicate diseases for centuries. The RNA vaccine has never been used on a large scale prior to the COVID-19 pandemic.

If you read above and understand the difference between conventional and RNA vaccines, please click "Next" to proceed.

*Note: All subjects were given the above description page, but subjects were randomized into 10 groups and distributed the below questions based on (1) receiving the conventional vaccine or the mRNA vaccine, and (2) a hypothetical percentage of people who had already received either of the vaccines in the respondent’s country. In the questions below, these options are marked by a “/”. Subjects only saw one of the options in the square brackets ( “[“ and “]” ), depending on which group they were in.*

Suppose that [a conventional vaccine / an RNA vaccine] for COVID-19 received approval for human use and is available for the public.

1. How much do you think the government should subsidize the [conventional / RNA] COVID-19 vaccine?

- 100%, You don't pay. It's free.
- 75%
- 50%
- 25%
- 0%, You pay for the vaccine yourself.

1. If the [conventional / RNA] COVID-19 vaccine were provided to you for free, how likely are you to accept the vaccination?

- Very likely
- Somewhat likely
- Neither likely nor unlikely
- Somewhat unlikely
- Very unlikely

1. Suppose the [conventional / RNA] COVID-19 vaccine is endorsed by your Government, free, [but no one / and 20% / and 40% / and 60% / and 80% of people] in your country [has / have] received the vaccine. How likely are you to accept the vaccination?

- Very likely
- Somewhat likely
- Neither likely nor unlikely
- Somewhat unlikely
- Very unlikely

1. Suppose the [conventional / RNA] COVID-19 vaccine is endorsed by your Government, free, [but no one / and 20% / and 40% / and 60% / and 80% of people] in your country has received the vaccine. How likely are you to recommend your loved ones to be vaccinated with this new vaccine?

- Very likely
- Somewhat likely
- Neither likely nor unlikely
- Somewhat unlikely
- Very unlikely

1. What is your age?
2. What is your gender?

- Male
- Female
- Other

1. Years spent in formal education:

- Up to 6 years
- 7-9 years
- 10-12 years
- over 12 years

1. What is your annual household income? *(Customized across countries)*

- Less than $20,000
- $20,000 to $34,999
- $35,000 to $49,999
- $50,000 to $74,999
- $75,000 to $99,999
- $100,000 or more

**Supplementary Discussion**

In total, 35,180 subjects responded to the online survey experiment. Seven responses were dropped from the analysis sample due to invalid entries for age. The final analysis sample is 35,173.

Supplementary Data 4 provides summary statistics of the subjects by the vaccine type to which they were randomly assigned. The average age of subjects was 39.2 years old, 50.0% of subjects were female, and 61.6% of subjects received more than 12 years of formal education.

We also surveyed participants’ attitudes towards vaccines in general: 44.2% strongly agreed that vaccines are safe; 45.1% strongly agreed that vaccines are effective; and 59.6% strongly agreed that vaccines are important for children to have. When asked about a friend’s child, 76.3% reported that it is very necessary for the child to be vaccinated. There were no significant differences in either the demographic variables or the attitudes towards vaccines in general between the conventional and mRNA vaccine groups.

However, there were significant differences in the attitudes towards Covid-19 vaccines: 75.1% reported that a conventional vaccine for Covid-19 should be entirely subsidized by the government, compared to 71.2% for an mRNA vaccine (two-sample proportions test; *z* = 8.375, *p* < 0.001, Cohen’s *h* = 0.089). Before being shown a hypothetical vaccine adoption rate in the country, 58.7% of respondents were very likely to accept a conventional vaccine for Covid-19 compared to 52.7% for an mRNA vaccine (two-sample proportions test; *z* = 11.274, *p* < 0.001, Cohen’s *h* = 0.120). The difference of 6 percentage points is statistically significant but relatively small in magnitude.

We then randomly assigned the respondents into one of five hypothetical vaccine adoption rates (0%, 20%, 40%, 60%, 80%). After being presented with a hypothetical adoption rate, on average, 52.1% reported that they were very likely to accept a conventional vaccine for Covid-19, compared to 48.7% for an mRNA vaccine (two-sample proportions test; *z* = 6.430, *p* < 0.001, Cohen’s *h* = 0.069). The difference of 3 percentage points is again statistically significant, but smaller in magnitude compared to the vaccine acceptance rate before being assigned a hypothetical vaccine adoption rate. We also asked whether they would recommend the vaccines to their loved ones. 50.4% said they are very likely to recommend a conventional vaccine for Covid-19, compared to 46.6% for an mRNA vaccine (two-sample proportions test; *z* = 6.985, *p* < 0.001, Cohen’s *h* = 0.075).

The general vaccine attitudes (i.e., belief that vaccines are safe, effective, and important for children to take) are highly correlated with COVID-19 vaccine acceptance. The correlation between COVID-19 vaccine acceptance (“very likely” to accept a COVID-19 vaccine) and strong agreement that vaccines are safe is 0.422 (*p* < 0.001); between vaccine acceptance and strong agreement that vaccines are effective is 0.393 (*p* < 0.001); between vaccine acceptance and strong agreement that vaccines are important for children is 0.372 (*p* < 0.001); and between vaccine acceptance and strong agreement that it is necessary for a friend to vaccinate the child is 0.368 (*p* < 0.001).

Supplementary Figure 1 shows the distribution of responses, by country, to four questions: (a) pooled willingness to accept a COVID-19 vaccine (either conventional or mRNA), (b) the extent to which they agree that vaccines are safe, (c) effective, and (d) important for children to take. Not surprisingly, we find that countries with more positive attitudes towards vaccines generally tend to have higher acceptance rates for Covid-19 vaccines.

**Supplementary Figure 1. Vaccine acceptance and general vaccine attitudes, by country. Brazil *n* = 3,799 responses, Mexico *n* = 3,819 responses, UK *n* = 3,745 responses, India *n* = 4,731 responses, Indonesia *n* = 3,758 responses, China *n* = 3,924 responses, USA *n* = 3,743 responses, Germany *n* = 3,878 responses, Russia *n* = 3,776 responses. Boxes indicate median and interquartile range (IQR) with the red dot specifying median value, and whiskers indicate range (data within 1.5*IQR).**


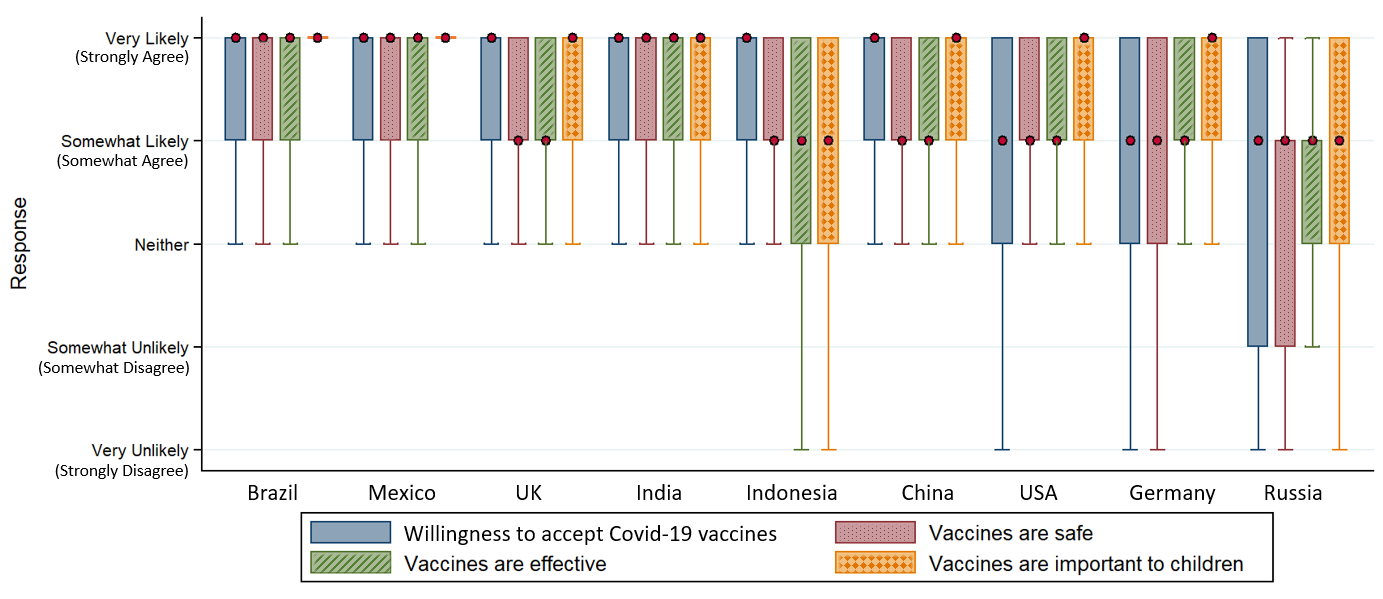


Supplementary Figure 2 shows the distribution of responses on the likelihood of vaccine acceptance, by age group. We observe an increasing relationship between vaccine acceptance and age. Subjects who were seventy years old or older were most willing to receive the Covid-19 vaccine: 67.5% were very likely to accept a conventional vaccine and 64.7% an mRNA vaccine. 62.5% of subjects in their sixties reported being very likely to accept a conventional vaccine and 54.9% being very likely to accept an mRNA vaccine. On the other hand, among subjects younger than sixty, only 49.3-52.3% were very likely to accept a conventional vaccine and 45.9-48.6% an mRNA vaccine. The positive relationship may be due to higher vulnerability to the disease among older subjects.

**Supplementary Figure 2. The distribution of responses on the likelihood of vaccine acceptance, by age group and by vaccine type. Conventional vaccine *n* = 17,527 responses (under 30 *n* = 4,910 responses, 30s *n* = 5,292 responses, 40s *n* = 3,281 responses, 50s *n* = 2,115 responses, 60s *n* = 1,356 responses, 70 and above *n* = 573 responses); mRNA vaccine *n* = 17,646 responses (under 30 *n* = 5,034 responses, 30s *n* = 5,324 responses, 40s *n* = 3,245 responses, 50s *n* = 2,134 responses, 60s *n* = 1,345 responses, 70 and above *n* = 564 responses). Boxes indicate median and interquartile range (IQR) with the red dot specifying median value, and whiskers indicate range (data within 1.5*IQR).**


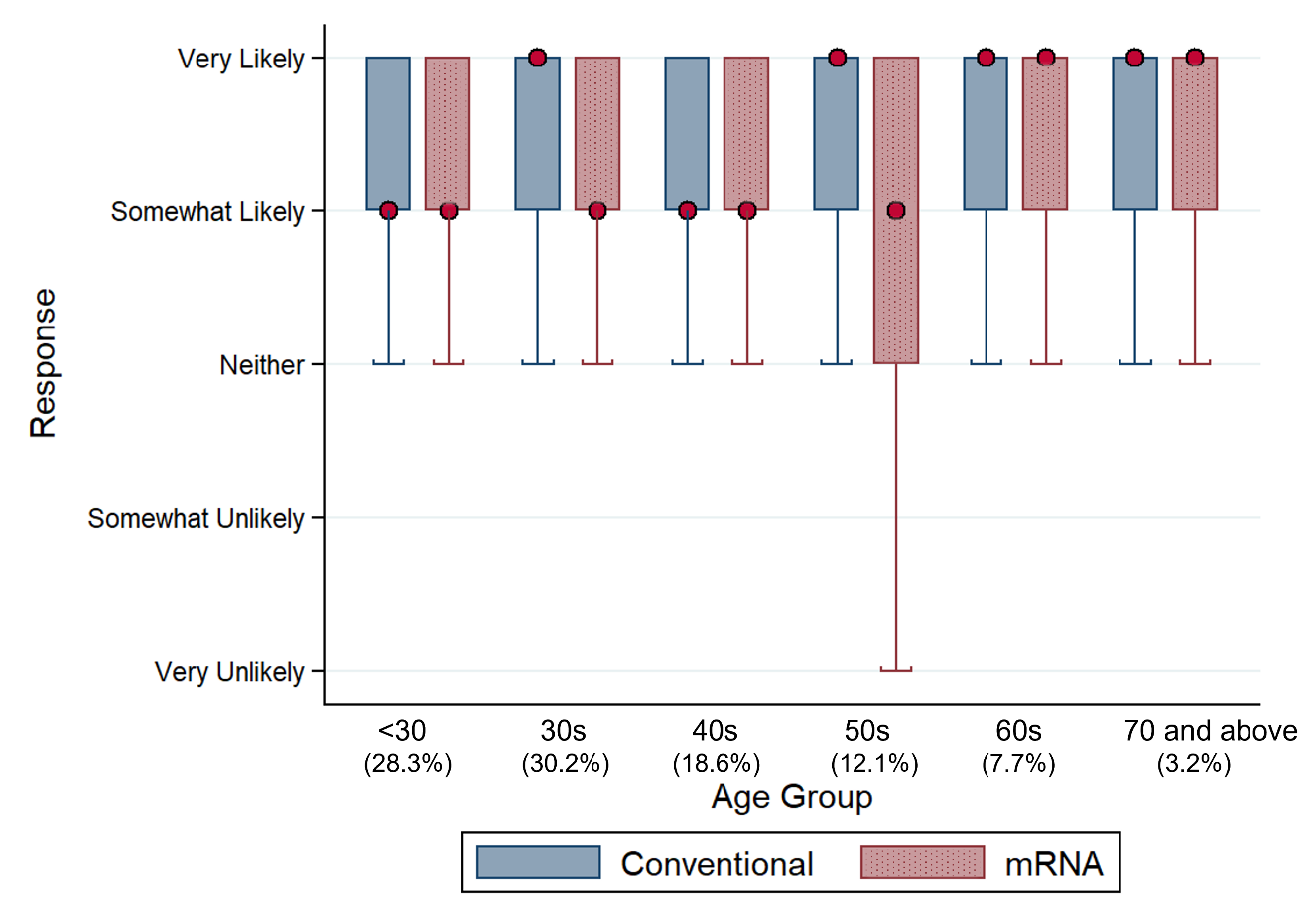


Supplementary Figure 3 shows the distribution of responses on the likelihood of vaccine acceptance, by years of formal education. We observe an increasing relationship between vaccine acceptance and years of formal education. Among respondents who received six or fewer years of formal education, 44.0% were very likely to receive a conventional vaccine for Covid-19 and 39.4% an mRNA vaccine. The acceptance rates were highest among respondents who received more than 12 years of formal education: 55.3% for conventional and 52.0% for mRNA vaccines.

**Supplementary Figure 3. The distribution of responses on the likelihood of vaccine acceptance, by years of formal education and by vaccine type. Conventional vaccine *n* = 17,527 responses (6 years or less *n* = 1,233 responses, 7-9 years *n* = 1,327 responses, 10-12 years *n* = 4,137 responses, more than 12 years *n* = 10,830 responses); mRNA vaccine *n* = 17,646 responses (6 years or less *n* = 1,209 responses, 7-9 years *n* = 1,340 responses, 10-12 years *n* = 4,269 responses, more than 12 years *n* = 10,828 responses). Boxes indicate median and interquartile range (IQR) with the red dot specifying median value, and whiskers indicate range (data within 1.5*IQR).**


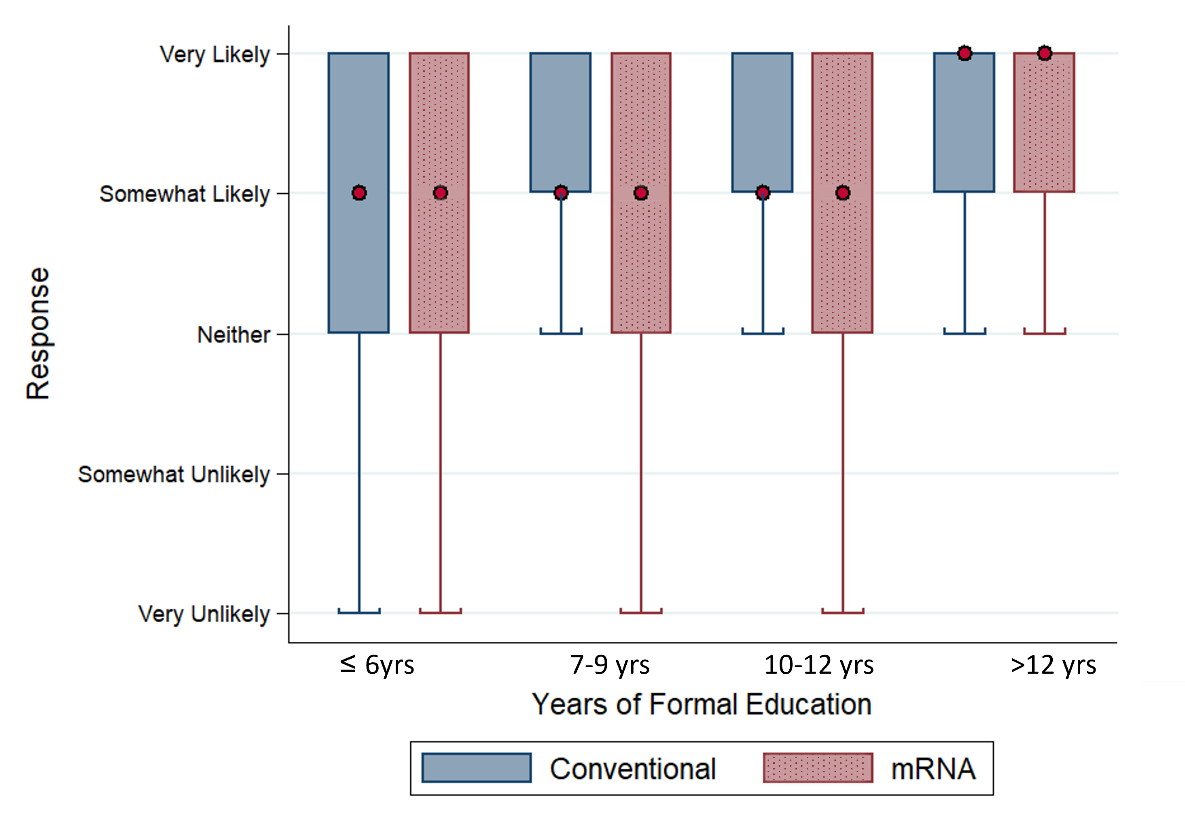


In the main text, we primarily focused on the proportion of people who reported that they were “very likely” to accept the Covid-19 vaccine. This was based on prior research which suggests that people often do not follow through with their mildly positive intentions to act.^1^ As a robustness check, we report results using the proportion of people who are either “very likely” or “somewhat likely” to accept the vaccine. This measure can be interpreted as an optimistic upper-bound of potential vaccine acceptance rates.

Overall, 79.5% of the respondents were either “very likely” or “somewhat likely” to accept a conventional vaccine for Covid-19, and 77.0% for an mRNA vaccine (two-sample proportions test, *z* = 5.584, *p* < 0.001, Cohen’s *h* = 0.060).

At a 0% adoption rate, only 65.2% are at least somewhat likely to accept the conventional vaccine, and 61.2% for an mRNA vaccine. At a 20% adoption rate, the proportions jump to 81.0% and 78.2% for the conventional and mRNA vaccines, respectively. Willingness continues to increase beyond the 20% adoption rate but at slower rates. At a 40% adoption rate, 82.5% and 80.8% were at least somewhat likely to accept the conventional and mRNA vaccines, respectively; at a 60% adoption rate, the proportions were 83.9% and 81.7%, respectively; and at an 80% adoption rate, the proportions were 84.7% and 83.5%, respectively. The results again suggest the importance of early vaccine adopters in increasing public acceptance of Covid-19 vaccines.

The gaps between mRNA and conventional vaccines are small, especially at higher vaccine adoption rates. We conducted two-sample proportions tests to assess the statistical significance of the gap, at each vaccine adoption rate. The results are as follows. At a 0% adoption rate, *z* = 3.483, *p* < 0.001; at a 20% adoption rate, *z* = 2.986, *p* = 0.003; at a 40% adoption rate, *z* = 1.876, *p* = 0.061; at a 60% adoption rate, *z* = 2.457, *p* = 0.014; and at an 80% adoption rate, *z* = 1.403, *p* = 0.161.

We conducted a two-way analysis of variance (ANOVA) to test the statistical significance of the reduced gap at higher adoption rates. There was a significant difference in the proportion of people who are at least somewhat likely to get vaccinated, between mRNA vaccines and conventional vaccines (F(1,35163) = 30.94, *p* < 0.001), as well as among adoption rates (F(4,35163) = 315.31, *p* < 0.001), but there was no interaction effect between the two treatment variables (F(4,35163) = 1.22, *p* = 0.298).

Supplementary Data 5 provides a full breakdown of the attitudes towards conventional and mRNA vaccines for each country. Interestingly, in China, 91.6% and 89.0% said they are at least somewhat likely to accept the conventional and mRNA vaccines, respectively. These rates are highest among the nine countries we surveyed, in contrast to China being sixth out of nine countries in the proportion of “very likely” responses. This is due to an unusually large proportion of “somewhat likely” responses in China. The high proportion of “somewhat likely” vaccine acceptance rates in China is consistent with earlier surveys.^2,3^

Six out of nine countries (China, Mexico, India, Brazil, Indonesia, UK) have more than 80% of respondents who are at least somewhat likely to be vaccinated for Covid-19. For these countries, convincing the people who are only mildly positive about receiving a Covid-19 vaccine would be key to achieving community immunity through inoculations.

On the other hand, three out of nine countries (Germany, USA, and Russia) had less than 80% who were somewhat or very likely to accept a Covid-19 vaccine. In Germany, 71.2% and 68.2% say they are at least somewhat likely to accept a conventional and an mRNA vaccine, respectively; in the USA, the rates are 69.3% and 66.0%, respectively; and in Russia, 58.6% and 53.1%, respectively. In these countries, it is not enough to simply convince the people who are mildly positive towards a Covid-19 vaccine; stronger measures must be taken to convince a significant portion of people who have neutral or negative attitudes towards the vaccine.

In Supplementary Data 6, columns (1)-(3), we test for the difference in the magnitude of the novelty penalty across gender, age, and the adoption rate in the country. We find no significant differences. In column (4), we observe heterogeneity in the magnitude of the novelty penalty across the nine countries. The novelty penalty was largest in in Russia, where the novel vaccine technology reduces the odds of higher vaccine acceptance by 24.2% (odds ratio 0.758, *p* < 0.001), followed by Brazil (odds ratio 0.776, *p* < 0.001), the UK (odds ratio 0.843, *p* = 0.008), the USA (odds ratio 0.852, *p* = 0.011), Indonesia (odds ratio 0.864, *p* = 0.020) and China (odds ratio 0.880, *p* = 0.033). The smallest novelty penalty was observed in Mexico (odds ratio 0.962, *p* = 0.557), India (odds ratio 0.903, *p* = 0.066) and Germany (odds ratio 0.891, *p* = 0.054).

Supplementary Table 1 reports *p*-values for the difference in novelty penalty between any two countries.

**Supplementary Table 1. P-Values for the Differences in Novelty Penalty Across Countries**

|  | **Russia**  **[OR=0.758]** | **Brazil**  **[OR=0.776]** | **UK**  **[OR=0.843]** | **USA**  **[OR=0.853]** | **Indonesia**  **[OR=0.864]** | **China**  **[OR=0.880]** | **Germany**  **[OR=0.891]** | **India**  **[OR=0.903]** | **Mexico**  **[OR=0.962]** |
| --- | --- | --- | --- | --- | --- | --- | --- | --- | --- |
| **Russia**  **[OR=0.758]** | - |  |  |  |  |  |  |  |  |
| **Brazil**  **[OR=0.776]** | 0.795 | - |  |  |  |  |  |  |  |
| **UK**  **[OR=0.843]** | 0.232 | 0.375 | - |  |  |  |  |  |  |
| **USA**  **[OR=0.853]** | 0.181 | 0.309 | 0.902 | - |  |  |  |  |  |
| **Indonesia**  **[OR=0.864]** | 0.131 | 0.238 | 0.776 | 0.870 | - |  |  |  |  |
| **China**  **[OR=0.880]** | 0.078 | 0.159 | 0.618 | 0.706 | 0.833 | - |  |  |  |
| **Germany**  **[OR=0.891]** | 0.056 | 0.122 | 0.524 | 0.604 | 0.725 | 0.885 | - |  |  |
| **India**  **[OR=0.903]** | 0.033 | 0.081 | 0.417 | 0.490 | 0.604 | 0.757 | 0.874 | - |  |
| **Mexico**  **[OR=0.962]** | 0.007 | 0.021 | 0.147 | 0.178 | 0.234 | 0.313 | 0.383 | 0.455 | - |

Notes: The table reports *p*-values for the difference in novelty penalty between any two countries. The countries are listed in descending order of the estimated magnitude of the novelty penalty (Column 4 of Supplementary Data 6). We obtain the *p*-value for a difference between countries *X* and *Y* by estimating a multivariate ordered logistic regression, with vaccine acceptance as the dependent variable, and the following independent variables: mRNA indicator, eight interactions of mRNA indicator and country indicators (eight countries excluding country *X*), gender, age, years of schooling, and country of residence. The estimated coefficient for the interaction of mRNA indicator and country *Y* indicator corresponds to the difference in novelty penalty between countries *X* (reference country) and *Y*. The *p*-value for this coefficient is reported in the table.

Finally, we examine the potential association between the urgency of the Covid-19 situation in the participants’ respective country and the degree of “novelty penalty”. The novelty penalty refers to the difference in vaccine acceptance rates between conventional and mRNA vaccines, i.e., the hesitation induced by the novelty of the vaccine technology. One simple measure of novelty penalty is the difference in the proportion of people very likely to be vaccinated between mRNA and conventional vaccines. For Covid-19 urgency, we use three measures: (a) the cumulative number of Covid-19 cases / population; (b) the cumulative number of Covid-19 deaths / cumulative number of Covid-19 cases; and (c) the cumulative number of Covid-19 deaths / 10,000 population. We use the cumulative number of Covid-19 cases and deaths as of February 28, 2021, which is approximately when the survey was implemented. The first measure indicates how widespread the virus is in the country, the second measure approximates how fatal the virus is conditional on contracting the virus in the country, and the third measure indicates the prevalence of deaths due to Covid-19 in the country.

Supplementary Figure 4A shows a scatter plot of the novelty penalty vs. cumulative number of Covid-19 cases per population for the nine countries. We find a positive correlation of 0.198, which is not statistically significant (*p* = 0.609). The data for the graphs is given in Supplementary Data 3.

**Supplementary Figure 4A. Scatter plot of novelty penalty vs. cumulative number of Covid-19 cases per population**


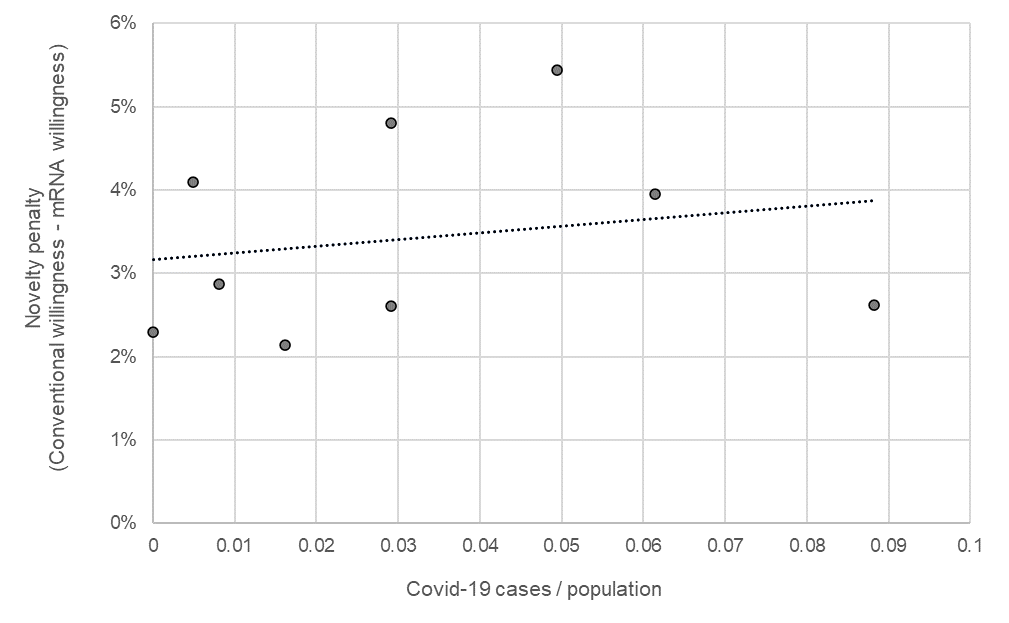


Supplementary Figure 4B shows a scatter plot of the novelty penalty vs. the ratio between the cumulative number of deaths and cases for the nine countries. A negative correlation of -0.488 indicates that, on average, countries with a higher case-fatality ratio tend to have lower novelty penalty. Again, the correlation coefficient is not found to be statistically significant (*p* = 0.183).

**Supplementary Figure 4B. Scatter plot of novelty penalty vs. cumulative number of Covid-19 deaths / cumulative number of Covid-19 cases**


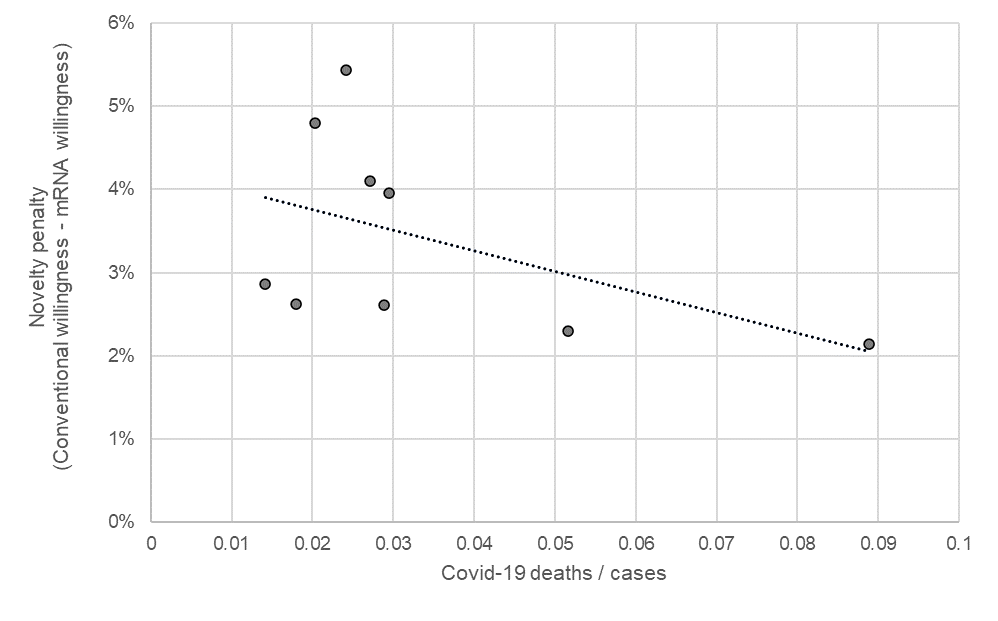


Finally, Supplementary Figure 4C shows a scatter plot of the novelty penalty vs. the cumulative number of Covid-19 deaths per 10,000 population. The estimated correlation coefficient is close to zero and is not statistically significant (*p* = 0.877).

**Supplementary Figure 4C. Scatter plot of novelty penalty vs. cumulative number of Covid-19 deaths / 10,000 population**


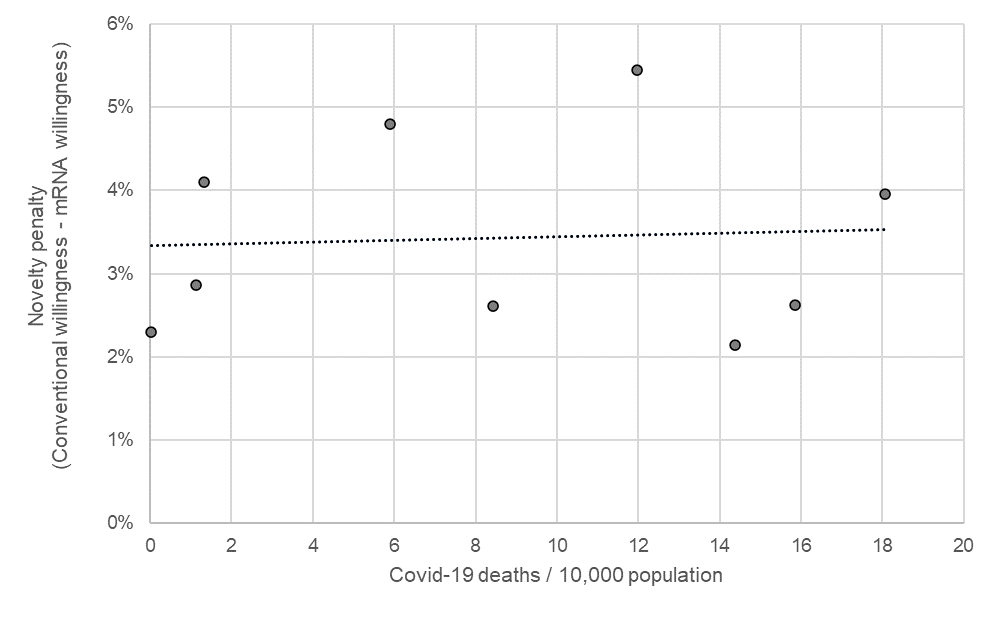


**Supplementary References**

1. Webb, T. L. and Sheeran, P. Does changing behavioral intentions engender behavior change? A meta-analysis of the experimental evidence. *Psychological Bulletin*, **132**(2), 249–268, 2006.
2. Lazarus, J. V., et al. A global survey of potential acceptance of a COVID-19 vaccine. *Nature Medicine* **27**, 225–228 (2021).
3. Ipsos. Global Attitudes on a COVID-19 Vaccine, 2020. https://www.ipsos.com/sites/default/files/ct/news/documents/2020-09/global-attitudes-on-a-covid-19-vaccine-ipsos-survey-for-wef-2020.pdf. Accessed on February 1, 2021.
